# The Putative RNA-Binding Protein Dri1 Promotes the Loading of Kinesin-14/Klp2 to the Mitotic Spindle and Is Sequestered into Heat-Induced Protein Aggregates in Fission Yeast

**DOI:** 10.3390/ijms22094795

**Published:** 2021-04-30

**Authors:** Masashi Yukawa, Mitsuki Ohishi, Yusuke Yamada, Takashi Toda

**Affiliations:** 1 Laboratory of Molecular and Chemical Cell Biology, Graduate School of Integrated Sciences for Life, Hiroshima University, 1-3-1 Kagamiyama, Higashi-Hiroshima, Hiroshima 739-8530, Japan; chonghuidashi858@gmail.com (M.O.); youliangshantian821@gmail.com (Y.Y.); takashi-toda@hiroshima-u.ac.jp (T.T.); 2 Hiroshima Research Center for Healthy Aging (HiHA), Hiroshima University, Hiroshima 739-8530, Japan

**Keywords:** fission yeast, kinesin-14, RNA-binding protein, mitotic spindle, heat stress

## Abstract

Cells form a bipolar spindle during mitosis to ensure accurate chromosome segregation. Proper spindle architecture is established by a set of kinesin motors and microtubule-associated proteins. In most eukaryotes, kinesin-5 motors are essential for this process, and genetic or chemical inhibition of their activity leads to the emergence of monopolar spindles and cell death. However, these deficiencies can be rescued by simultaneous inactivation of kinesin-14 motors, as they counteract kinesin-5. We conducted detailed genetic analyses in fission yeast to understand the mechanisms driving spindle assembly in the absence of kinesin-5. Here, we show that deletion of the *dri1* gene, which encodes a putative RNA-binding protein, can rescue temperature sensitivity caused by *cut7-22*, a fission yeast kinesin-5 mutant. Interestingly, kinesin-14/Klp2 levels on the spindles in the *cut7* mutants were significantly reduced by the *dri1* deletion, although the total levels of Klp2 and the stability of spindle microtubules remained unaffected. Moreover, RNA-binding motifs of Dri1 are essential for its cytoplasmic localization and function. We have also found that a portion of Dri1 is spatially and functionally sequestered by chaperone-based protein aggregates upon mild heat stress and limits cell division at high temperatures. We propose that Dri1 might be involved in post-transcriptional regulation through its RNA-binding ability to promote the loading of Klp2 on the spindle microtubules.

## 1. Introduction

During mitosis, cells assemble a bipolar spindle formed by microtubules (MTs) emanating from two spindle poles, thereby ensuring balanced chromosome segregation. For this reason, the spindle architecture must be built up accurately, which is driven by several motor and non-motor MT-associated proteins (MAPs) [1,2,3]. Among them, kinesin motors play significant roles in organizing spindle MTs. Particularly, evolutionarily conserved kinesin-5 proteins are vital for bipolar spindle formation [4,5]. Activity of kinesin-5 is under control of mitotic kinases, including CDK1 [6,7,8,9,10,11]. During mitosis, kinesin-5 motors crosslink the overlapping antiparallel ends of interpolar MTs and then slide them apart via plus end-directed motility; this results in the generation of outward forces pushing and separating newly duplicated centrosomes (spindle pole bodies, SPBs, in yeast). Kinesin-5 is also crucial for stabilizing the overlapping MT array at the anaphase spindle midzone and for promoting effective spindle elongation. The spindle defect caused by loss of kinesin-5-mediated outward forces depends on opposite inward forces supplied by minus end-directed kinesin-14 motors, which pull spindle poles together. Accordingly, the simultaneous inactivation of kinesin-14 motors neutralizes kinesin-5 deficiency, highlighting force balance’s importance in proper spindle formation.

In fission yeast, kinesin-5/Cut7 is also crucial for cell growth, and the deletion or the mutation of *cut7* causes a mitotic arrest with characteristic V-shaped monopolar spindle MTs [12,13]. The lethality derived from Cut7 malfunction is suppressed by the deletion of kinesin-14s, Pkl1 and Klp2, which generate collaborative inward forces against Cut7 in a spatially distinct manner [14,15,16,17,18,19,20,21,22]. Pkl1 forms a ternary complex with the other two cofactors Msd1 and Wdr8 (referred to as the MWP complex) and is predominantly localized at mitotic SPBs; Klp2 is localized mainly along the spindle MT [16,23].

Recent studies have provided evidence that a large number of RNA-binding proteins (RBPs) are present on mitotic spindles and involved in microtubule regulation and mitotic spindle formation [24]. Treatment with transcription inhibitors or RNase disrupts the mitotic spindle structure independent of active translation [25,26], implying that functional mRNAs or non-coding RNAs (ncRNAs) act as regulators or structural components of the mitotic spindle. Despite several studies on the regulation or function of RBPs [27,28,29], more RBPs and RNAs required for spindle formation are deemed to await identification.

It is known that most RBPs assemble non-membrane-enclosed granules with mRNAs and ncRNAs in the cytoplasm through liquid–liquid phase separation [30]. These RNA granules include stress granules (SGs) and processing bodies (P bodies). SGs are formed upon various environmental stress conditions and serve as reservoirs for translationally stalled mRNAs [31]. By contrast, P bodies are present under basal conditions and associate with mRNA metabolism, such as mRNA degradation and nonsense-mediated mRNA decay [32]. Recent studies in fission yeast show that several heat shock chaperones assemble protein aggregate centers (PACs) upon mild heat stress [33,34]. PACs are proposed to act as a seed for the nucleation of SGs at severer high-temperature stress, sequester misfolded proteins and protect them from degradation, thereby promoting their effective refolding upon relief from heat stress [35].

We previously isolated *skf* (*skf*: *s*uppressor of *k*inesin *f*ive) mutants as genetic suppressors of the *cut7-22* temperature-sensitive (ts) growth defect to understand the molecular mechanisms of spindle assembly in the absence of kinesin-5 in fission yeast [36]. These mutations consist of 10 linkage groups (*skf1*–*skf10*), in which *skf1*–*skf6* were categorized into two functional groups. One group (*skf1*–*skf3*) comprises genes encoding the components of the MWP complex (Skf1/Pkl1, Skf2/Wdr8 and Skf3/Msd1) [16], consistent with the previous reports [14,15,16,17]. The other group (*skf4*–*skf6*) includes genes encoding tubulins (Skf4/Nda3/β-tubulin and Skf5/Atb2/α2-tubulin) [37] and a non-motor MAP (Skf6/Mal3/EB1) [38]. These mutations lead to the destabilization of the spindle MTs and consequently reduce the Klp2 intensities. Hence, the rescue of *cut7* mutants by these suppressors is derived from the quantitative down-regulation of inward force provided by Klp2 activity [36].

In this study, we have found that *skf7*, the genetic identity of which remained unknown in our previous study [36], is allelic to *dri1*, which encodes a putative RBP containing canonical RNA-binding domains such as the RNA recognition motif (RRM). We show that the truncation or mutation of the RNA-binding domains within Dri1, and the deletion of this gene, rescues the *cut7* ts phenotype. Detailed analyses of the cells lacking Dri1 demonstrate that the amount of kinesin-14/Klp2 proteins on the spindle is significantly decreased in the absence of Dri1 function. We also show that Dri1 is localized to the cytoplasm throughout the cell cycle. Intriguingly, upon mild heat stress, a portion of Dri1 assembles into PACs [33,34]. We propose that PACs limit the functionality of Dri1 under heat stress conditions by spatially sequestering it into the cytoplasmic aggregates.

## 2. Results

### 2.1. Loss of Function of a Putative RNA Binding Protein Dri1/Skf7 Suppresses the Cut7-22 Temperature-Sensitive Growth Defect

**Figure 1:**
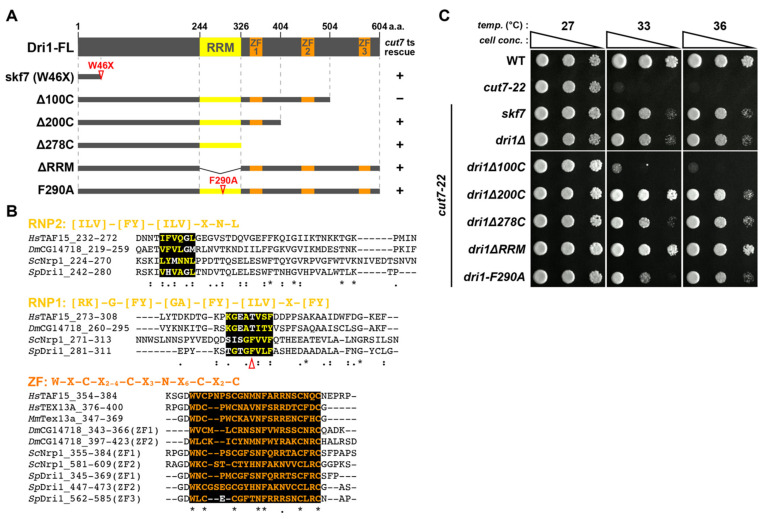
Systematic truncation and mutation analysis of Dri1 highlights the importance of RRM and RanBP2-type ZF domains. (**A**) Schematic representation of full-length Dri1 (Dri1-FL), *skf7* mutant (W46X), C-terminal truncation mutants (Δ100C, Δ200C, and Δ278C), an RRM truncation mutant (ΔRRM) and an RRM point mutant (F290A). The plus or minus sign indicates “rescue” or “no rescue”, respectively, of the *cut7-22* ts phenotype by each *dri1* mutant. (**B**) Dri1 is a member of the Tex13 protein family and is conserved across different species. Amino acid sequences from the indicated species have been aligned using the Clustal W. Consensus amino acids for the RRM (RNP1 and RNP2) or the RanBP2-type zinc finger domain (ZF) are highlighted in yellow or orange letters on black background, respectively. The conserved sequences are highlighted using symbols (asterisk represents identical residues; colon represents highly conservative residues; period represents moderately conservative residues). The F290A mutation is shown with an open arrowhead. Positions of amino acids used for *H. sapiens* TEX13A (376–400), *M. musculus* Tex13a (347–369), *D. melanogaster* CG14718 (219–295, 343–366 and 397–423), *S. cerevisiae* Nrp1 (224–313, 355–384 and 581–609), *S. pombe* Dri1 (242–311, 345–369, 447–473 and 562–585). GenBank accession codes are as follows: *Hs*TEX13A, NP_001278206.1; *Mm*Tex13a, NP_080745.2; *Dm*CG14718, NP_650107.1; *Sc*Nrp1, NP_010114.1; *Sp*Dri1, NP_593574.1. The sequences of *H. sapiens* TAF15 (232–308, 354–384) obtained from GenBank (NP_631961.1) are also aligned as reference sequences. (**C**) Spot test. Indicated strains were serially (10-fold) diluted, spotted onto rich YE5S plates and incubated at various temperatures for 2–3 d.

To explore the genetic network that plays a role in conferring the non-essentiality of kinesin-5, we attempted to identify genes responsible for suppressor mutations, *skf7*–*skf10*, which remained unassigned in our previous study [36]. To this end, we conducted the next generation sequencing approach and succeeded in identifying a mutation site of *skf7*. This mutation is a substitution of an amino acid residue at position 46 from tryptophan to a stop codon (W46X) within a putative RBP Dri1 (Figure 1A,B). Consistent with this assignment, the deletion of the *dri1* gene suppressed the ts phenotype of *cut7-22* at 36 °C (Figure 1C). We also addressed whether the *dri1* deletion suppresses ts phenotypes of other *cut7* mutant alleles (*cut7-21*, *cut7-23*, *cut7-24* and *cut7-446*) [12,13,18]. Interestingly, *dri1Δ* effectively rescued the ts phenotype of *cut7-21* at 36 °C, whereas it was incapable of rescuing other *cut7* alleles (Supplementary Figure S1), indicating that the suppression is allele-specific.

### 2.2. The RNA-Binding Domains of Dri1 Are Required for Its Function

According to a comprehensive database for the fission yeast (Pombase; www.pombase.org (accessed on 21 April 2021)), Dri1 has two types of putative RNA-binding domains: one is the RRM in its central region and the other is a RanBP2-type zinc finger domain comprising three repeats (ZF1–3) in its C-terminus (Figure 1A). The HCOP database (https://www.genenames.org/tools/hcop/ (accessed on 21 April 2021)) also assigns Dri1 as a member of the testis-expressed gene 13 protein (Tex13) family including human TEX13A, mouse Tex13a, fruit fly CG14718 and budding yeast Nrp1, which have multiple ZFs [39,40] (Figure 1B). The RRM is the most common RNA-binding motif that contains two highly conserved short-sequence blocks known as RNP1 and RNP2 [41]. This motif is conserved among the TEX13A members in the fruit fly and yeast (Figure 1B).

To assess the functional significance of these domains, we constructed strains containing a series of truncated or point-mutated *dri1* (Figure 1A) and examined the suppression of the *cut7-22* ts phenotype. Truncation of C-terminal 100 amino acids (Δ100C) had no effect, but truncation of C-terminal 200 and 278 amino acids (Δ200C, Δ278C) suppressed the ts growth defect at 36 °C (Figure 1A,C), suggesting that ZF1 and ZF2 are essential for the Dri1 function. Moreover, both the truncation and mutation of the RRM domain (ΔRRM and F290A) suppressed the *cut7-22* ts phenotype (Figure 1A,C). We found that these truncated/mutated Dri1 proteins were normally expressed, as demonstrated by immunoblotting (Supplementary Figure S2). Thus, these findings imply that the RNA-binding ability of Dri1 is essential for its function.

### 2.3. Dri1 Is Required for Proper Localization of Kinesin-14/Klp2 on the Spindle Microtubules

**Figure 2:**
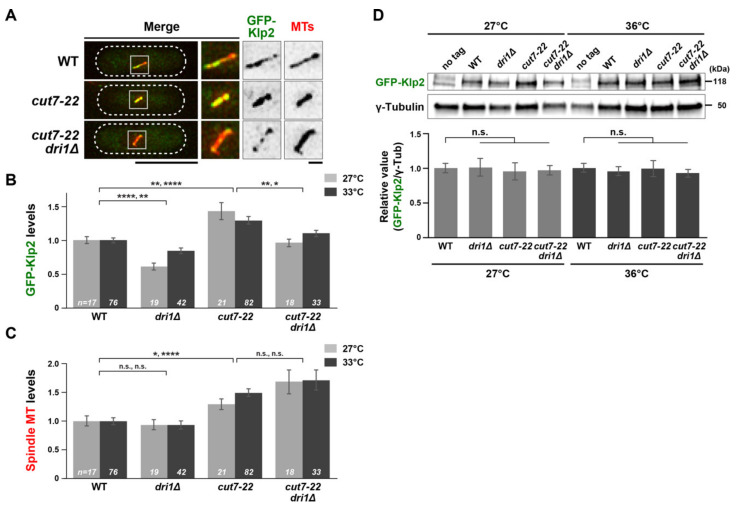
Dri1 is required for the Klp2 localization on the spindle microtubules. (**A**) Representative images showing mitotic localization of GFP-Klp2 on the spindle microtubule are presented in indicated cells. All strains contain GFP-Klp2 and mCherry-Atb2 (a microtubule marker). Cells were incubated at 27 °C. The cell peripheries are outlined with dotted lines, and areas containing spindle microtubules (squares) are enlarged in the three panels on the right-hand side. Scale bars, 10 μm (bottom left) and 1 μm (bottom right). (**B**,**C**) Quantification of GFP-Klp2 levels on the spindle microtubule (**B**) and spindle microtubules (**C**). Individual strains shown in (**A**) were grown at 27 °C, and a half of each culture was shifted to 33 °C, while the remaining half was kept at 27 °C. Fluorescence intensities were measured 2 h later. Then, 33 °C was used as the restrictive temperature, as fluorescence signals of GFP-Klp2 were quenched rapidly at 36 °C, which made quantitative measurement of GFP-Klp2 signal intensities difficult. The total values of GFP-Klp2 fluorescence intensities were divided by the spindle length in each cell. The values obtained from wild-type cells incubated at 27 °C and 33 °C were set as 1 and compared with those from other strains incubated at the same temperature. All *p*-values were obtained from the two-tailed unpaired Student’s *t*-test. Data are presented as the means ± SE (≥17 cells). *, *p* < 0.05, **, *p* < 0.01, ****, *p* < 0.0001, n.s., not significant. (**D**) Quantification of the total levels of GFP-Klp2 protein in whole-cell extracts. Indicated strains containing GFP-Klp2 and a control non-tagged strain were grown at 27 °C or 36 °C for 2 h, and protein extracts were prepared, followed by immunoblotting with anti-GFP and anti–γ-tubulin antibodies. Relative amounts of each GFP-Klp2 protein were normalized using those of γ-tubulin as a control. Results are given as the means ± SE (*n* = 5). The relative amounts obtained from wild-type cells incubated at 27 °C and 36 °C were set as 1 and compared with those from other strains incubated at the same temperature. Data sets were compared with a two-tailed unpaired Student’s *t*-tests (n.s., not significant).

We previously showed that in the *cut7-22* mutant, intensities of spindle MTs are significantly increased, by which more kinesin-14/Klp2 proteins accumulate on the spindles, resulting in generating excessive inward force and inhibiting bipolar spindle formation [20,36]. We measured the levels of Klp2 on pre-anaphase spindle MTs in *dri1Δ* or *cut7-22dri1Δ* mutant cells to compare with those in wild-type or *cut7-22* cells and investigated whether *dri1*/*skf7* mutations rescue the *cut7* ts mutants through the downregulation of kinesin-14/Klp2 function. Interestingly, we found that the deletion of *dri1* significantly reduced Klp2 levels at 27 °C and 33 °C (Figure 2A,B). We then measured the intensities of pre-anaphase spindle MTs (<3 μm). Unlike the Klp2 proteins, the levels of spindle MTs in *cut7-22dri1Δ* cells remained similar to those of *cut7-22* mutant cells and higher than those of the wild-type cells (Figure 2A,C). The reduction in Klp2 intensities on the spindle MTs could not be attributed to the reduction in its protein levels, as the deletion of *dri1* did not affect the total amount of the Klp2 protein (Figure 2D). Taken together, we posit that Dri1 promotes the loading of Klp2 to the spindle MTs, which counteracts Cut7 function in bipolar spindle formation.

### 2.4. Dri1 Is Dispensable for the Spindle Localization of EB1/Mal3

Previous reports showed that the EB1 plus-end tracking protein interacts with kinesin-14 in several organisms, including fission yeast, and that this interaction is essential for efficient spindle assembly [42,43,44,45]. Given this notion, one possibility is that Dri1 is involved in loading Klp2 on the spindle MTs through the regulation of EB1/Mal3. To address this notion, we measured the Mal3 protein levels. However, the Mal3 protein levels did not change in the presence or absence of Dri1 (Supplementary Figure S3A). Similarly, the levels of Mal3-GFP on the spindle MTs were almost the same both in wild-type and *dri1Δ* cells (Supplementary Figure S3B). These results indicate that Dri1 promotes the Klp2 loading onto the spindle MTs without affecting the Mal3 levels on the spindle.

### 2.5. Cytoplasmic Dri1 Counteracts the Kinesin-5/Cut7 Function

**Figure 3:**
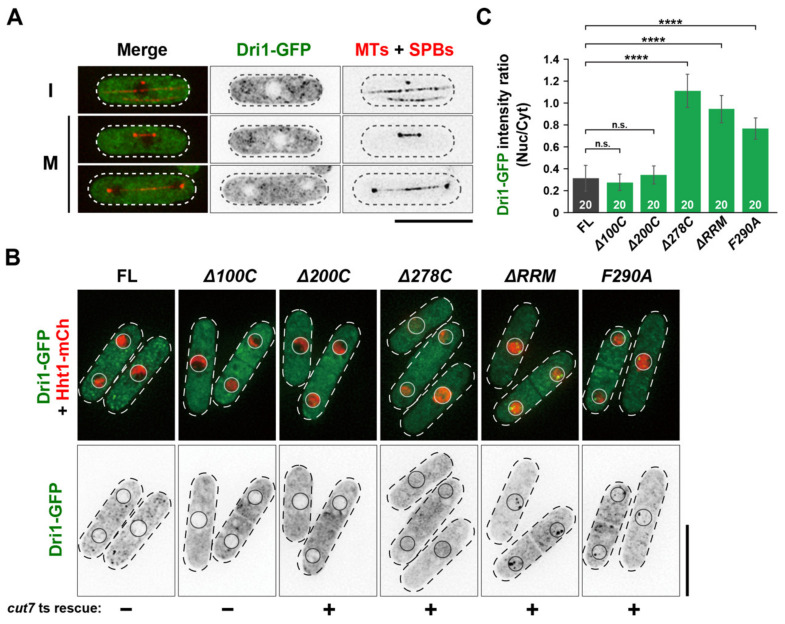
Cytoplasmic localization of Dri1 depends on the RNA recognition motif and RanBP2-type zinc finger domains. (**A**) Dri1 is localized in the cytoplasm throughout the cell cycle. Localization of Dri1-GFP during interphase (I) or mitosis (M) is shown. Cells were grown in rich media at 27 °C. Microtubules are visualized by mCherry-Atb2, while the SPB was marked with Sid4-mRFP [46]. (**B**) The putative RNA-binding domains of Dri1 are essential for its export from the nucleus to the cytoplasm. Cells expressing the GFP-tagged full-length or various truncated/mutated Dri1 (Dri1-GFP) together with the chromosome marker Hht1-mCherry (Histone H3 h3.1) [47,48] were grown in rich media at 27 °C, and their localization was observed. (**C**) The bar graph shows quantification of nuclear enrichment of Dri1-GFP signal. Fold enrichment of Dri1-GFP signal in the nucleus (nucleus/cytoplasm) was quantified. All *p*-values were obtained from the two-tailed unpaired Student’s t test. Data are presented as the means ± SE (20 cells). ****, *p* < 0.0001, n.s., not significant. The cell peripheries are indicated with dotted lines. Scale bars, 10 μm.

In order to find clues for the function of Dri1, we examined the subcellular localization of endogenous Dri1-GFP integrated into the chromosome under the native promoter in wild-type cells. The Dri1-GFP signal is present in the cytoplasm in a dispersed manner throughout the cell cycle and appears to be excluded from the nucleus (Figure 3A). Next, we observed the localization of the truncated/mutated Dri1-GFP proteins. Dri1Δ100C-GFP and Dri1Δ200C-GFP signals showed the same localization pattern as the full-length protein, whereas other truncated/mutated Dri1-GFP signals are found in both cytoplasmic and nuclear sides (Figure 3B,C), correlating with the suppression of the *cut7-22* ts growth defect (see Figure 1A,C). These results highlight the importance of the RRM and two ZFs (ZF1 and ZF2) for the proper localization and function of Dri1.

### 2.6. Dri1 Is Exported from the Nucleus in a Rae1-Dependent Manner

A subset of RBPs, particularly those binding mRNA, is exported from the nucleus and locates to the cytoplasm by a transport factor Rae1 [49,50,51]. Accordingly, *rae1-167* ts mutants exhibit defective mRNA export and rapidly accumulate poly(A)^+^ RNA and mRNA-binding proteins in the nucleus at the restrictive temperature [49,52,53]. Given this notion and the existence of RNA-binding motifs within Dri1, we investigated the subcellular localization of Dri1-GFP in the *rae1-167* mutant cells. In *rae1-167* cells grown at the permissive temperature (27 °C), Dri1-GFP localized to the cytoplasm, as in wild-type cells (Supplementary Figure S4A). Notably, when cells were shifted to the restrictive temperature (36 °C), Dri1-GFP was accumulated in the nucleus, more specifically in the chromatin region in *rae1-167* cells, whereas Dri1 remained in the cytoplasm in wild-type cells (Supplementary Figure S4A and S4B). Thus, Dri1 is likely to be dynamically shuttled between the nucleus and the cytoplasm and exported from the nucleus directly or indirectly through the Rae1-dependent mRNA export pathway (Supplementary Figure S4C).

### 2.7. Dri1 Assembles into Protein Aggregate Centers upon Mild Temperature Shift-Up

**Figure 4:**
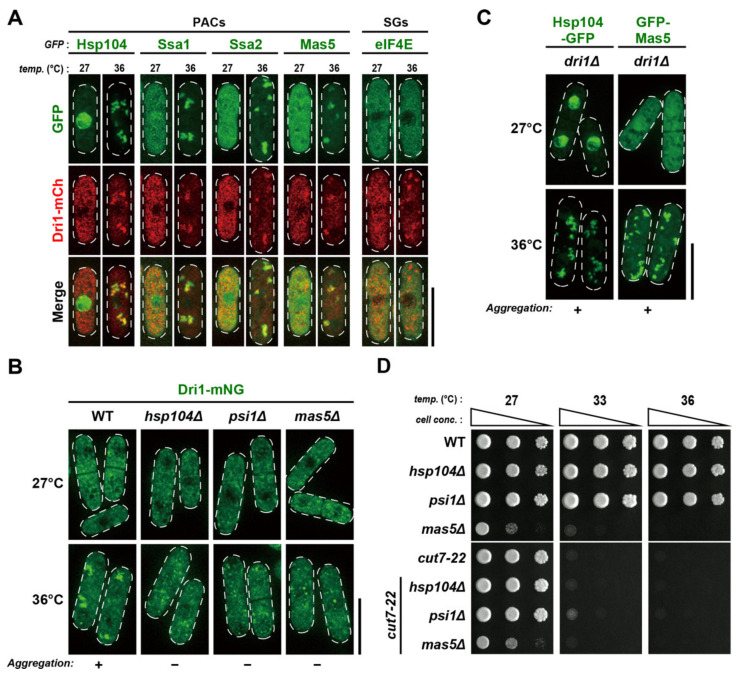
Dri1 is present at protein aggregate centers upon mild temperature shift-up. (**A**) A portion of the Dri1 protein co-localizes with the components of PACs by temperature shift-up. Cells containing Dri1-mCherry and different GFP-tagged chaperones (Hsp104, Ssa1, Ssa2 and Mas5) were grown at 27 °C and then shifted to 36 °C for 2 h. SGs are not formed under this condition, as monitored in cells co-expressing Dri1-mCherry and eIF4E-GFP (an SG marker) after incubation at 36 °C for 2 h. (**B**) Several chaperones, such as Hsp104, Psi1 and Mas5, are required for the Dri1 assembly during mild temperature shift-up. Wild-type, *hsp104Δ*, *psi1Δ* and *mas5Δ* strains expressing Dri1-mNeonGreen were grown at 27 °C and then shifted to 36 °C for 2 h. (**C**) Dri1 is dispensable for the assembly of PACs. The *dri1Δ* strain expressing Hsp104-GFP or GFP-Mas5 was grown at 27 °C and then was shifted to 36 °C for 2 h. (**D**) Spot test. Indicated strains were serially (10-fold) diluted, spotted onto rich YE5S plates and incubated at various temperatures for 2–3 d. The cell peripheries are indicated with dotted lines. Scale bars, 10 μm.

We noted that during previous experiments using *rae1-167*, the Dri1-GFP protein in wild-type cells aggregated in the cytoplasm after shift-up to 36 °C for 2 h, although a significant amount of Dri1-GFP still existed in the cytoplasm without aggregation (Supplementary Figure S4A). Recently, it was reported that some misfolded proteins collapse into PACs upon mild temperature shift-up, e.g., 36 °C, thereby being protected from degradation [33,34]. We monitored the co-localization of Dri1 and known PAC markers (several chaperones including Hsp104, Hsp16, Hsp70s/Ssa1 and Ssa2, Hsp90/Swo1 and Hsp40s/Psi1 and Mas5) to address whether Dri1 is incorporated into PACs. The simultaneous expression of Dri1-mCherry and each GFP-tagged chaperone in cells revealed an almost complete co-localization after shift-up to 36 °C for 2 h (Figure 4A and Supplementary Figure S5), indicating that Dri1 is present at PACs. Conversely, we did not detect at this temperature any foci of the translation initiation factor eIF4E, a marker of SGs that are formed only under severer heat stress (>42 °C) [34,54] (Figure 4A).

We then examined the localization dependency between Dri1 and the chaperones. As shown in Figure 4B, the absence of Hsp104, Psi1 or Mas5 significantly impaired the formation of Dri1-mNeonGreen foci at 36 °C. By contrast, the lack of Dri1 had almost no impact, if any, on the assembly of Hsp104-GFP or GFP-Mas5 at 36 °C (Figure 4C). We next asked whether the lack of PACs per se suppressed the *cut7-22* ts phenotype. However, growth assays showed that the deletion of either of *hsp104*, *psi1* or *mas5*, which is necessary for PAC assembly [34], did not suppress *cut7-22* (Figure 4D). We, therefore, conclude that Dri1 is functional at 36 °C without localizing to PACs.

### 2.8. PACs Spatially and Functionally Sequester Dri1 upon Mild Heat Stress

**Figure 5:**
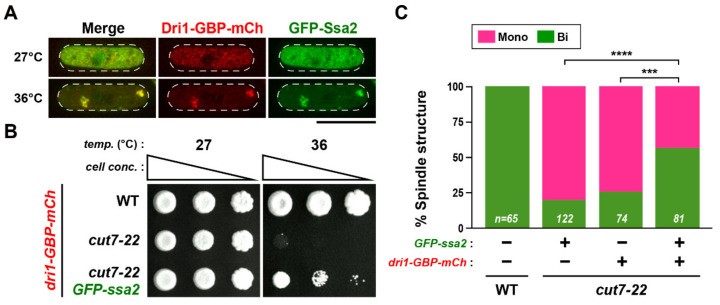
Forced tethering of Dri1 to PACs is sufficient to rescue *cut7-22*. (**A**) Visualization of Dri1-GBP-mCherry localization in strains in the presence of GFP-Ssa2. Indicated strains were grown at 27 °C and then shifted to 36 °C for 2 h. The cell peripheries are indicated with dotted lines. Scale bar, 10 μm. (**B**) Spot test. Indicated strains were serially (10-fold) diluted, spotted onto rich YE5S plates and incubated at 27 °C and 36 °C for 5 d. (**C**) *cut7-22* cells containing Dri1-GBP-mCherry alone, GFP-Ssa2 alone or both were grown at 27 °C and shifted to 36 °C for 2 h. The percentage of cells with either mono- (magenta) or bipolar spindles (green) was counted. For comparison, the data for wild-type cells grown under the same condition are also shown. The sample numbers (n) for individual strains are indicated on the bottom of columns. All *p*-values were obtained from the two-tailed χ^2^ test. ***, *p* < 0.001, ****, *p* < 0.0001.

It was recently shown that the Pyp1 protein phosphatase, a negative regulator of the stress-activated Sty1/Spc1 MAP kinase, localizes to PACs, leading to activation of this MAP kinase pathway by physically dissociating Pyp1 from Sty1 [33]. Given this result, we hypothesized that Dri1 would be spatially sequestered by PACs upon heat stress, thereby causing the down-regulation of its function for bipolar spindle assembly. If this is the case, further enforced tethering of Dri1 to PACs might be sufficient to rescue the *cut7* ts phenotype. To assess this possibility, we implemented the GFP–GFP-binding protein (GBP) protein targeting system [55] and constructed a *cut7-22* strain containing Dri1-GBP-mCherry and GFP-tagged PACs-localizing Ssa2. As shown in Figure 5A, tethering Dri1 to PACs was successful at 36 °C. Intriguingly, a spot test of these strains indicated that forced recruitment of Dri1 to PACs rescued, albeit partially, the *cut7* ts growth defect at 36 °C; note that *cut7-22* mutant cells containing only Dri1-GBP-mCherry in the absence of GFP-Ssa2 were still ts (Figure 5B). Indeed, these Dri1-tethered cells could form bipolar spindles at this high temperature; however, consistent with the spot test, the suppression of *cut7-22* was incomplete, as we still observed a certain fraction (~40%) of cells exhibiting monopolar spindles (Figure 5C). Hence, a simple recruitment of Dri1 to PACs substantially inhibits the functionality of Dri1, indicating that Dri1 may be spatially sequestered by PACs upon heat stress, leading to down-regulation.

### 2.9. Dri1 Accumulates at Stress Granules and Limits the Maximum Temperature for Growth

**Figure 6:**
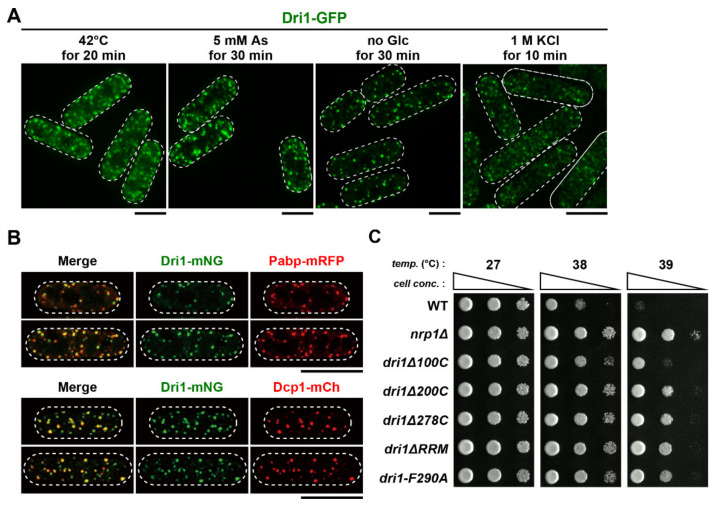
Dri1 accumulates at stress granules and limits the maximum temperature for growth. (**A**) Dri1 rapidly translocates to the cytoplasmic aggregates under various stress conditions. Cells expressing Dri1-GFP were grown in rich media at 27 °C and then exposed to various stresses. (**B**) Dri1 co-localizes with an SG marker poly(A)-binding protein, Pabp and a P body marker, Dcp1 under glucose depletion condition. Cells expressing either Pabp-mRFP or Dcp1-mCherry, together with Dri1-mNeonGreen, were grown in rich media at 27 °C and then shifted to glucose-depleted medium at 27 °C for 30 min. (**C**) Malfunction of Dri1 acquires tolerance to high temperature stress. Indicated strains were serially (10-fold) diluted, spotted onto rich YE5S plates and incubated at various temperatures for 2–3 d. The cell peripheries are indicated with dotted lines. Scale bars, 10 μm.

As PACs serve as a seed for the nucleation of SGs under severe heat shock [34], we wondered that Dri1 might localize to SGs in response to various environmental stresses. To check this notion, we determined the subcellular localization of Dri1 under several stress conditions. Upon exposure to thermal stress (42 °C) for 20 min, small patches of granule-like structures were observed throughout the cytoplasm (Figure 6A). Intriguingly, exposure of cells to arsenite, a reagent well-known to induce SGs in both yeast and mammalian cells [31,56,57,58,59], also induced the Dri1-positive granules (Figure 6A). Additionally, Dri1-positive granules were formed in cells treated with glucose-depleted media (no Glc for 30 min) and hyperosmotic stress (1 M KCl for 10 min) (Figure 6A). The Dri1-positive granules co-localized with the SG markers including the poly(A)-binding protein, Pabp and the P body marker, Dcp1 under glucose-deprived conditions [60,61] (Figure 6B).

To examine the possible functions of Dri1 under stresses, we checked the growth of the *dri1Δ* cells exposed to various stress conditions. We found that the *dri1Δ* cells acquired tolerance to high temperature, as they could form colonies at 39 °C, whereas wild-type cells could not (Figure 6C). In line with this result, other truncated/mutated *dri1* mutants were also capable of growing at 39 °C. As shown in Supplementary Figure S6, microscopic observation of cells incubated at 39 °C showed that while >50% of wild-type cells ceased cell division with wider cell shape containing thick septa, *dri1Δ* cells committed cell separation but then a fraction of these cells underwent cell lysis. Thus, it appears that Dri1 limits cell division at high temperatures (e.g., 39 °C) to protect cells from unfavorable lysis upon cell separation. In contrast to cell division at higher temperatures, resistance against other stresses was not acquired in the *dri1Δ* cells (Supplementary Figure S7). Thus, by localizing to SGs, Dri1 may play another role besides regulating kinesin-14/Klp2 function. 

## 3. Discussion

In this study, we explore why the loss of function of the Dri1 protein rescues the *cut7* ts phenotype. We have found that the *dri1* deletion lessens Klp2 localization on the mitotic spindle, which would account for the suppression of *cut7* ts mutants. We also show that the RNA-binding ability of this protein is required for proper cytoplasmic localization and function. Our findings suggest that Dri1 promotes the loading of Klp2 on the spindle MTs through its RNA-binding ability. Interestingly, under heat stress conditions, Dri1 is spatially sequestered into PACs. Although the biological significance of this sequestration remains to be determined, we assume that PACs-localizing Dri1 physically dissociates from a cytoplasmic pool, leading to temporal pause of the Dri1-mediated RNA processing at high temperatures.

### 3.1. Dri1 Promotes Loading of Kinesin-14/Klp2 onto Spindle MTs

**Figure 7:**
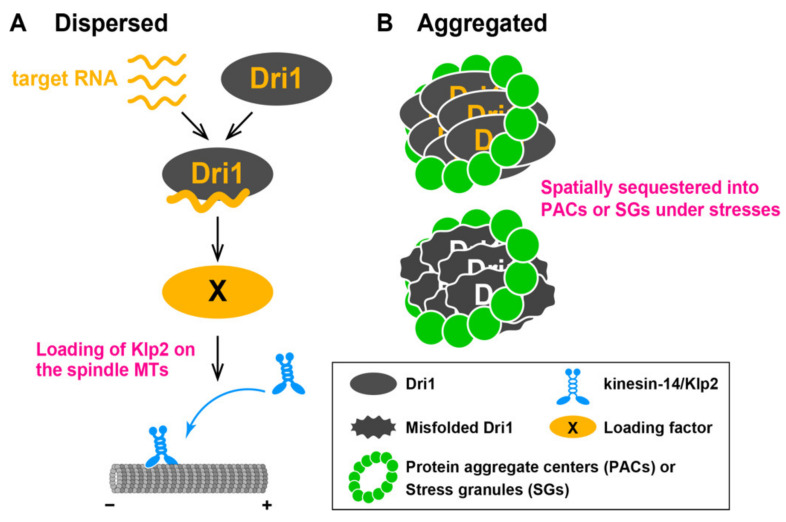
Schematic model of the role for Dri1 in bipolar spindle assembly and growth at high temperatures. (**A**) Under normal growth condition, Dri1 displays dispersed cytoplasmic patterns and acts to load kinesin-14/Klp2 on the spindle microtubules through its RNA-binding activity. Dri1 is shuttled between the cytoplasm and the nucleus by means of its RNA-binding activity. Dri1 might be involved in the processing of its target mRNA, which encodes an unidentified loading factor (X) of Klp2 onto the spindles. (**B**) Under mild heat stress, Dri1 is spatially sequestered by PACs, thereby leading to down-regulation of its function in bipolar spindle assembly. Dri1 is also important for limiting cell growth at 39 °C. In the absence of Dri1, cells manage to divide at 39 °C; however, these cells are not healthy and tend to undergo lysis upon cell separation. At higher temperature (42 °C) or under other adverse stresses, Dri1 is incorporated into SGs. Dri1 proteins localizing to PACs and SGs might be misfolded, by which they bind chaperones that are components of these aggregates. For simplicity, the role of Dri1 involving heterochromatin formation via histone deacetylase and RNA interference pathways [62] is not included in this figure.

Unlike other *skf4*–*skf6* mutants, the *dri1* deletion reduced the amount of Klp2 on the mitotic spindles without affecting the stability of spindle MTs or the total levels of Klp2 protein. We show that in the *dri1* deleted cells, Mal3, a known loading factor of Klp2 [42], normally localizes to the spindle. A previous study reported that the fission yeast septation initiation network (SIN) kinase, Sid2 phosphorylates Klp2, by which phosphorylated Klp2 cannot interact with Mal3, leading to prevention from its loading onto spindle MTs [42]. It is, thus, possible that Dri1 promotes the loading of Klp2 to the spindle MTs through SIN-Sid2. Alternatively, Dri1 may accelerate the nuclear import of Klp2, which is a prerequisite for Klp2 loading to the mitotic spindle. Although, currently, the mechanism of Dri1-mediated Klp2 loading to the mitotic spindle remains unsolved, we presume that Dri1 is involved in post-transcriptional regulation of its target mRNA, which encodes an unidentified loading factor (X) of Klp2 onto the mitotic spindle (Figure 7A).

### 3.2. How Does Dri1 Play a Role through Its RNA-Binding Ability? 

Our results support the hypothesis that Dri1 interacts with RNA molecules through its RNA-binding domains and plays a role in RNA-mediated processes, as the Dri1 proteins containing either truncation or mutation in these domains cannot function properly. We posit that Dri1 is exported together with its target RNA from the nucleus in a Rae1-dependent manner, thereby regulating the RNA processing in the cytoplasm. We, however, do not exclude the possibility that some fraction of Dri1 is retained in the nucleus and may have some function. In line with this notion, very recently, it has been reported that Dri1 physically interacts with Dbp4, the DNA polymerase epsilon, and is required for heterochromatin silencing at pericentromeres [62]. In this work, Dri1 is shown to interact with the pericentromeric heterochromatin transcripts and act in the RNA interference-mediated heterochromatin pathway. Other target RNA molecules of Dri1 remain currently unknown. Therefore, it is crucial to identify them to clarify further function of Dri1 in the future research.

### 3.3. Identification of Dri1 as a Component of Protein Aggregate Centers and Stress Granules under Heat Stress

What is the physiological significance of the translocation of Dri1 into PACs and SGs in response to thermal stress? Our data indicate that the localization of Dri1 to PACs upon mild heat stress is not required for its counteracting role against Cut7. Rather, the GBP-GFP entrapment experiments suggest that PACs-localizing Dri1 is spatially sequestered from the cytoplasm, resulting in functional inhibition: suppressing the *cut7-22* ts mutant (Figure 7B). Intriguingly, the *dri1* deletion enhances tolerance against thermal stress; the *dri1Δ* cells are capable of cell division at high temperature (39 °C). We envision that cells control the Dri1-mediated RNA processing to modulate growth rate and/or cell division by which they could adapt to varied environmental conditions such as temperature changes. The mechanism of cell division at high temperatures and the rationale for heat resistance in the absence of Dri1 function are future issues to be explored.

### 3.4. Conservation of the Tex13/Dri1 Protein Family

The *dri1* homolog, the *Tex13* gene, was initially identified as one of the X-linked genes expressed only in mouse male germ cells, and the human ortholog, *TEX13A,* is also expressed exclusively in testes [63]. Both human TEX13A and mouse Tex13a contain a RanBP2-type ZF domain in their C-terminal region. Moreover, human TEX13A can bind to single-strand RNAs containing the sequence AGGUAA, thereby potentially regulating mRNA processing [64]. Interestingly, like fission yeast Dri1, mouse Tex13a is shown to be shuttled between the nucleus and the cytoplasm [65]. Intriguingly, budding yeast Nrp1 accumulates in SGs during glucose deprivation [66]. Although it is unclear whether yeast Dri1/Nrp1 proteins regulate transcription through mRNA processing, it would be of great interest to examine whether vertebrate TEX13 proteins localize to SGs upon various stresses and how this localization is regulated.

To conclude, this is the first study demonstrating that Dri1 plays a regulatory role in mitotic spindle assembly and heat stress response. Given the remarkable conservation of the Tex13 protein family, a similar mechanism may operate in the loading of kinesin-14 on the spindle MTs and a growth limitation at high temperatures in other eukaryotes. Further functional and molecular characterization of Dri1 and its target RNAs may help us to understand how eukaryotic cells regulate these two processes.

## 4. Materials and Methods

### 4.1. Strains, Media, and Genetic Methods

Fission yeast strains used in this study are listed in Supplementary Table S1. Media, growth conditions, and manipulations were performed as previously described [67]. For most of the experiments, rich YE5S liquid media and agar plates were used. Spot tests were performed by spotting 5–10 μL of cells at a concentration of 2 × 10^7^ cells/ml after 10-fold serial dilutions onto rich YE5S plates. Some of the YE5S plates also contained Phloxine B, a vital dye that accumulates in dead or dying cells and stains the colonies dark pink due to a reduced ability to pump out the dye. Plates were incubated at various temperatures from 27 °C to 39 °C as necessary.

### 4.2. Next Generation Sequencing and Annotation of skf7 Genes

To identify a mutation site of *skf7* strain, we performed the next generation sequencing analysis as we described previously [36].

### 4.3. Stress Treatment

Prior to stress treatment, the cells were grown to mid-log phase at 27 °C. Heat shock was imposed by transferring the culture tubes to a water bath at 42 °C for the indicated time. To the culture medium, 100 mM sodium arsenate dibasic heptahydrate (Katayama Chemical Industries Co., Ltd., Osaka, Japan) stock solution, 5.0 M KCl (Kanto Chemical Co., Inc., Tokyo, Japan, Cat. No. 32326-00) stock solution, Sorbitol (Kishida Chemical Co., Ltd., Osaka, Japan, Cat. No. 000-73236) and Hydrogen peroxide solution with concentration of 30% (wt/vol) (Santoku Chemical Industries Co. Ltd., Tokyo, Japan, CAS No. 7722-84-1) were added at the indicated concentrations. For glucose deprivation, cells were collected by centrifugation, washed in media lacking glucose and briefly centrifuged. Cells were then re-suspended in media lacking glucose and incubated at 27 °C for the indicated time.

### 4.4. Preparation and Manipulation of Nucleic Acids

Enzymes were used as recommended by the suppliers (New England Biolabs Inc. Ipswich, MA, USA and Takara Bio Inc., Shiga, Japan).

### 4.5. Strain Construction, Gene Disruption and Epitope Tagging

A PCR-based gene-targeting method [68,69] was used for complete gene disruption and epitope tagging (e.g., GFP, mNeonGreen, mCherry and GBP-mCherry) in the C terminus, by which all the tagged proteins were produced under the endogenous promoter.

### 4.6. Construction of Truncated/Mutated dri1 Mutants

To construct a series of C-terminally truncated Dri1-GFP, the GFP-kanR cassette was integrated at various positions as shown in Figure 1A (+326, +404 and +504) by a PCR-based gene-targeting method [68,69]. To construct the RRM-truncated or point-mutated Dri1, the *dri1-GFP-kanR* fragments were first amplified with PCR, and, using this fragment as a template, the truncated or point-mutated constructs within the RRM domain of Dri1 corresponding to *dri1ΔRRM-GFP-kanR* and *dri1-F290A-GFP-kanR*, respectively, were further amplified by two-step PCR. Individual DNA fragments were then transformed into wild-type cells (513, Supplementary Table S1) to replace the wild-type *dri1* gene with each construct. Note that all these mutant proteins were produced under the endogenous promoter.

### 4.7. Western Blot Analysis

Protein extracts were prepared by mechanical disruption of cells in an extraction buffer (50 mM Tris-HCl, pH 7.4, 1 mM EDTA, 150 mM NaCl, 0.05% NP-40, 10% glycerol, 1.5 mM p-nitrophenyl phosphate, 1 mM phenylmethylsulfonyl fluoride, and protease inhibitor cocktail obtained from Sigma-Aldrich, St. Louis, MO, USA) with acid-washed glass beads in a Multi-Beads Shocker (ON: 60 s, OFF: 60s, 2500 rpm, ×4 times, at 4 °C; Yasui-kikai, Osaka, Japan). Extracts were cleared of debris by centrifugation for 1 min and subsequently for 5 min at 13000 g. Protein concentrations were determined with a Bradford assay kit (Bio-Rad Laboratories, Inc., Richmond, CA, USA). Protein extracts were boiled in Laemmli buffer for 5 min and loaded and resolved on denaturing 4–12% gradient gels (Bio-Rad Laboratories, Inc., Richmond, CA, USA) and transferred onto polyvinylidene fluoride membranes. The membranes were blocked with 10% skim milk and then blotted with either anti-GFP antibody (rabbit polyclonal, TP401; Torrey Pines Biolabs, Houston, TX, USA) or anti-γ-tubulin antibody (mouse monoclonal, GTU-88; Sigma-Aldrich, St. Louis, MO, USA) at a dilution of 1:1000 in 1% skim milk. After having been washed, the blots were incubated with anti–rabbit or anti–mouse horseradish peroxidase–conjugated secondary antibody (GE Healthcare, Chicago, IL, USA) in 1% skim milk at a dilution of 1:2000. The ECL chemiluminescence kit (GE Healthcare, Chicago, IL, USA) was used for detection. Original images of Western blots are shown in Supplementary Figure S8.

### 4.8. Fluorescence Microscopy

Fluorescence microscopy images were obtained by using a DeltaVision microscope system (DeltaVision Elite; GE Healthcare, Chicago, IL, USA) that included a wide-field inverted epifluorescence microscope (IX71; Olympus, Tokyo, Japan) and a Plan Apochromat 60×, NA 1.42, oil immersion objective (PLAPON 60 × O; Olympus Tokyo, Japan). DeltaVision image acquisition software (softWoRx 6.5.2; GE Healthcare, Chicago, IL, USA) equipped with a charge-coupled device camera (CoolSNAP HQ2; Photometrics, Tucson, AZ, USA) was used. To keep cultures at the appropriate temperature, a temperature-controlled chamber (Air Therm SMT; World Precision Instruments Inc., Sarasota, FL, USA) was used. Images were taken as 14–16 sections along the z axis at 0.2 μm intervals; they were then deconvolved and merged into a single projection. Captured images were processed with Photoshop CS6 (version 13.0; Adobe, San Jose, CA, USA).

### 4.9. Quantification of Fluorescent Signal Intensities

For quantification of Dri1-GFP located at the nucleus and the cytoplasm, 14–16 sections were taken along the z-axis at 0.2-μm intervals. After deconvolution and projection images of maximum intensity, a 5 × 5-pixel (0.54-μm square) region of interest (ROI) with maximum sum intensity was determined. After subtracting the mean intensity of three regions outside cells as background intensities, the values of the maximum sum intensities were used for the calculation of the ratio of nuclear to cytoplasmic Dri1-GFP signal. For quantification of GFP-Klp2 and mCherry-Atb2 located on the metaphase spindle (spindle length < 3.0 μm), maximum sum intensity along individual spindles was determined, and after background subtraction, the values were divided by the spindle length in each cell.

### 4.10. Statistical Data Analysis

We used the two-tailed unpaired Student’s *t*-test to evaluate the significance of differences in different strains, unless otherwise stated. All the experiments were performed at least twice. Experiment sample numbers used for statistical testing were given in the corresponding figures and/or legends. We used this key for asterisk placeholders to indicate *p*-values in the figures: e.g., ****, *p* < 0.0001.

## Data Availability

The data that support the findings of this study are available from the corresponding author upon reasonable request.

## Supplementary Materials

The following are available online at https://www.mdpi.com/article/10.3390/ijms22094795/s1.

## Abbreviations

MT	Microtubule
MAP	Microtubule-associated protein
SPB	Spindle pole body
RBP	RNA-binding protein
ncRNA	Non-coding RNA
SG	Stress granule
P body	Processing body
PAC	Protein aggregation center
ts	Temperature sensitive
RRM	RNA recognition motif
ZF	Zinc finger
GBP	GFP-binding protein
