# Supplementary material for: The Putative RNA-Binding Protein Dri1 Promotes the Loading of Kinesin-14/Klp2 to the Mitotic Spindle and Is Sequestered into Heat-Induced Protein Aggregates in Fission Yeast
Source: Int J Mol Sci. 2021 Apr 30;22(9):4795. doi: 10.3390/ijms22094795 (PMC8125374; doi:10.3390/ijms22094795)
Supplement: Supplementary file 1 [file ijms-22-04795-s001.zip › ijms-1163877-supplementary.pdf]

International Journal of
Molecular Sciences

MDPI

Supplementary Materials

The Putative RNA-Binding Protein Dri1 Promotes the Loading of Kinesin-14/Klp2 to the Mitotic Spindle and Is Sequestered into Heat-Induced Protein Aggregates in Fission Yeast

Masashi Yukawa [1,2,*], Mitsuki Ohishi [1], Yusuke Yamada [1], and Takashi Toda [1,2]

Supplementary Figure S1: Suppression of the ts phenotype of *cut7* by *dri1Δ* is allele-specific.

Supplementary Figure S2: Various truncated/mutated Dri1 proteins are expressed normally like the full-length protein.

Supplementary Figure S3: Dri1 is dispensable for the Mal3 localization on the spindle microtubules.

Supplementary Figure S4: Dri1 is exported from the nucleus in a Rae1-dependent manner.

Supplementary Figure S5: A portion of the Dri1 protein co-localizes with other PAC components upon mild temperature shift-up.

Supplementary Figure S6: Characteristics of cell division in wild-type and *dri1Δ* cells grown at 39°C.

Supplementary Figure S7: Sensitivity/resistance of the *dri1* mutants is not altered under various stress conditions besides high temperature.

Supplementary Figure S8: Full scans of original images.

Supplementary Table S1: Fission yeast strains used in this study.

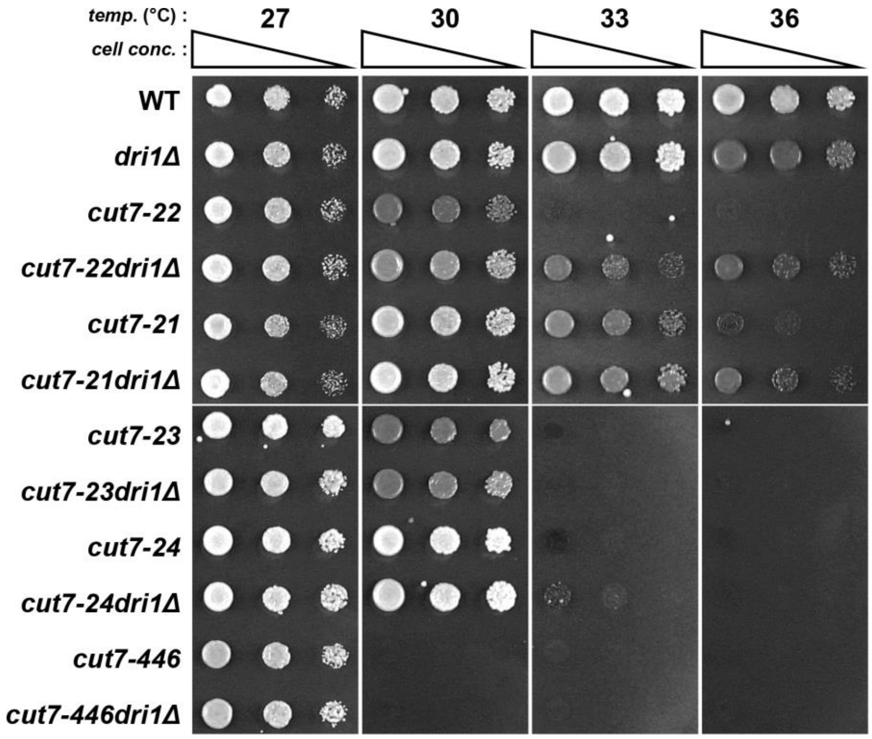

Supplementary Figure S1. Suppression of the ts phenotype of *cut7* by *dri1Δ* is allele-specific. Indicated strains were spotted onto rich YE5S agar plates and incubated at various temperatures for 2–3 days. 10-fold serial dilutions were performed in each spot.

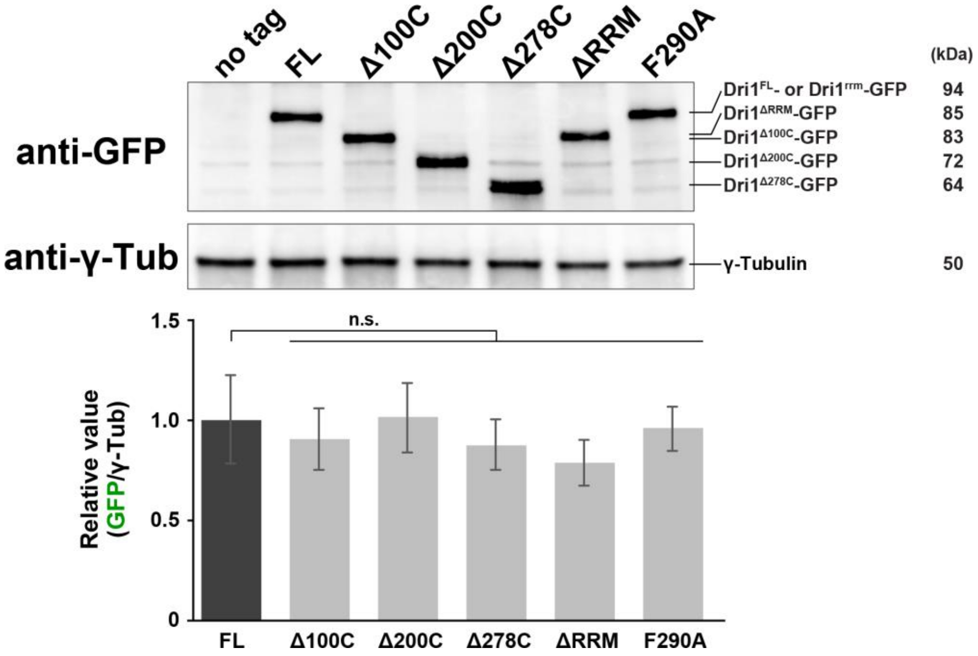

Supplementary Figure S2. Various truncated/mutated Dri1 proteins are expressed normally like the full-length protein. Quantification of the total levels of the full-length and various truncated/mutated Dri1-GFP proteins in whole-cell extracts was performed. Indicated strains containing Dri1-GFP and a control non-tagged strain were grown at 27 °C and protein extracts were prepared, followed by immunoblotting with anti-GFP and anti–γ-tubulin antibodies. Relative amounts of each Dri1-GFP protein were normalized using those of γ-tubulin as a control. Results are given as the means ± SE (n = 4). The relative amounts obtained from wild-type cells incubated at 27 °C were set as 1 and compared to those from other strains incubated at the same temperature. Data sets were compared with a two-tailed unpaired Student's t-tests (n.s., not significant).

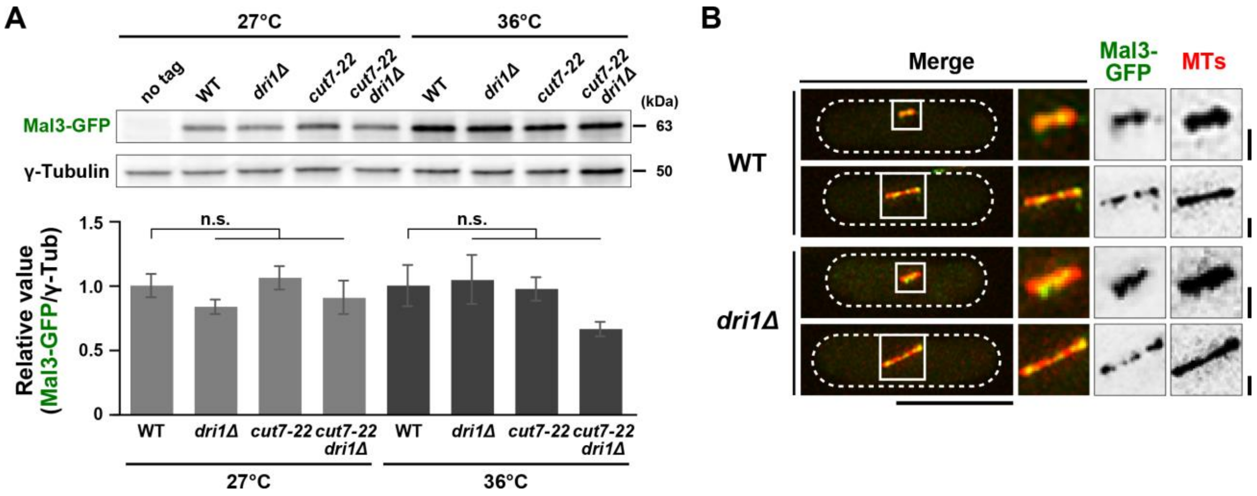

Supplementary Figure S3. Dri1 is dispensable for the Mal3 localization on the spindle microtubules. (**A**) Quantification of the total levels of Mal3-GFP protein in whole-cell extracts. Indicated strains containing Mal3-GFP and a control non-tagged strain were grown at 27 °C or 36 °C for 2h, and protein extracts were prepared, followed by immunoblotting with anti-GFP and anti–γ-tubulin antibodies. Relative amounts of each Mal3-GFP protein were normalized using those of γ-tubulin as a control. Results are given as the means ± SE (n = 2). The relative amounts obtained from wild-type cells incubated at 27 °C and 36 °C were set as 1 and compared to those from other strains incubated at the same temperature. Data sets were compared with a two-tailed unpaired Student's t-tests (n.s., not significant). (**B**) Representative images showing mitotic localization of Mal3-GFP on the spindle microtubule are presented in wild-type and *dri1Δ* cells. Both strains contain Mal3-GFP and mCherry-Atb2. Cells were incubated at 27 °C. The cell peripheries are outlined with dotted lines and areas containing spindle microtubules (squares) are enlarged in the three panels on the right-hand side. Scale bars, 10 μm (bottom left) and 1 μm (right).

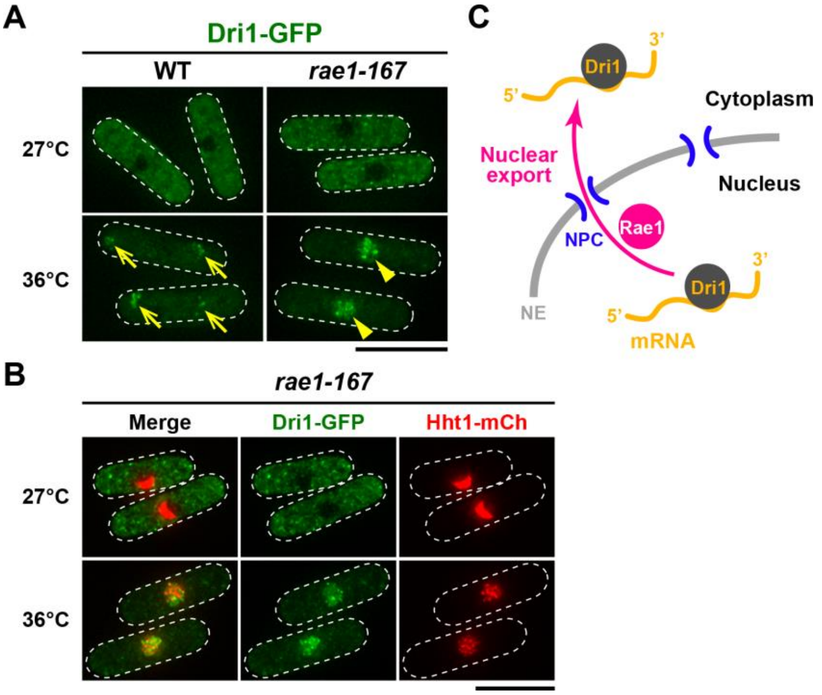

Supplementary Figure S4. Dri1 is exported directly or indirectly from the nucleus in a Rae1-dependent manner. (**A**) Wild-type and *rae1-167* cells containing Dri1-GFP were grown at 27 °C and then shifted to 36 °C for 2 h. The cytoplasmic aggregates formed at 36 °C in wild-type cells are pointed with yellow arrows, while the nuclear foci formed in *rae1-167* cells are shown with yellow arrowheads. (**B**) *rae1-167* cells containing Dri1-GFP and the chromatin marker Hht1-mCherry (Histone H3 h3.1) were grown at 27 °C and then shifted to 36 °C for 2 h. (**C**) A schematic illustration representing the export of Dri1 from the nucleus to the cytoplasm through the Rae1-dependent mRNA export pathway. The cell peripheries are indicated with dotted lines. Scale bars, 10 μm.

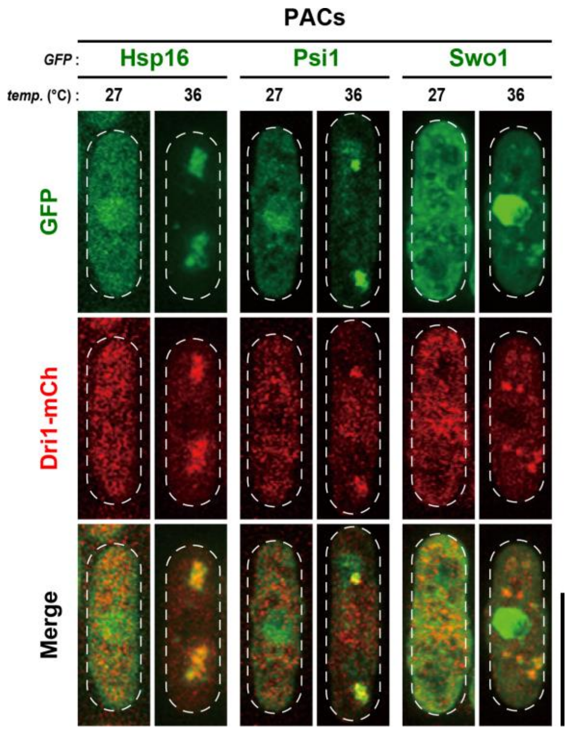

Supplementary Figure S5. A portion of the Dri1 protein co-localizes with other PACs components upon mild-temperature shift-up. Co-localization of Dri1-mCherry and different GFP-tagged chaperones (Hsp16, Psi1/Hsp40 and Swo1/Hsp90) after incubation at 36 °C for 2 h. The cell peripheries are indicated with dotted lines. Scale bar, 10 μm.

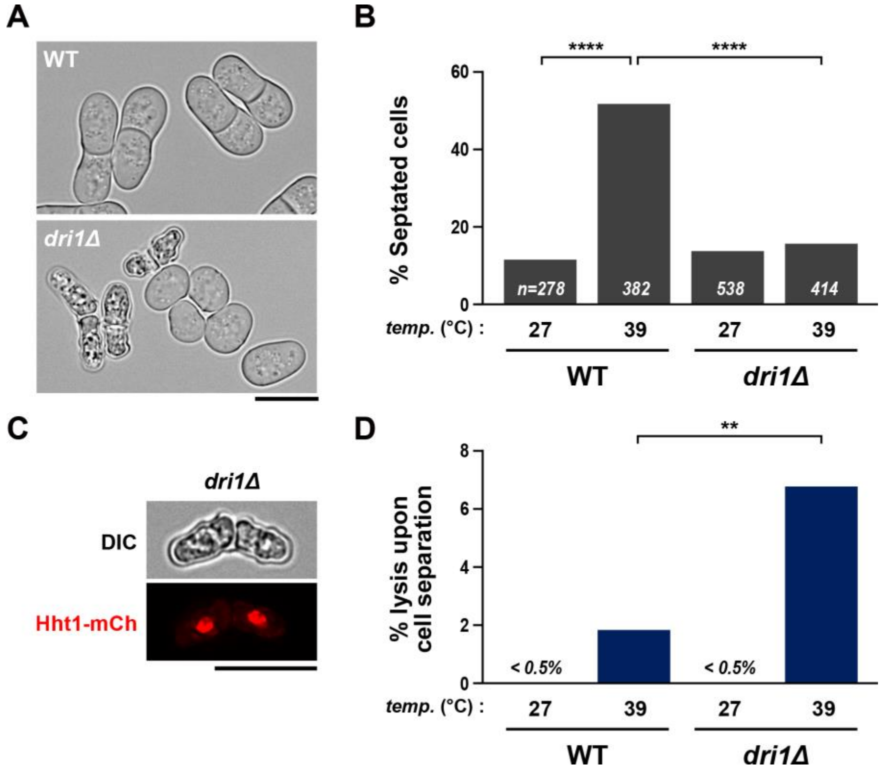

Supplementary Figure S6. Characteristics of cell division in wild-type and *dri1Δ* cells grown at 39 °C. (**A**) Wild-type and *dri1Δ* cells grown at 27 °C were shifted to 39 °C and incubated for 12 h. Representative images of cell morphologies are shown (top, wild-type; bottom, *dri1Δ*). (**B**) The percentage of cells containing septa in wild-type and *dri1Δ* cells grown at 27 °C or 39 °C. The sample size (n) is shown on the bottom of each column. (**C**) Representative images of *dri1Δ* cells displaying lysis upon cell separation at 39 °C. *dri1Δ* cells containing Hht1-mCherry (Histone H3 h3.1) grown at 39 °C were imaged (top, DIC; bottom Hht1-mCherry). (**D**) The percentage of cells displaying the cell lysis phenotype upon cell separation. Cells used in (**C**) were also counted in this figure. All *p*-values were obtained from the two-tailed χ^2 test. **, $p < 0.01$, ****, $p < 0.0001$, Scale bars, 10 μm.

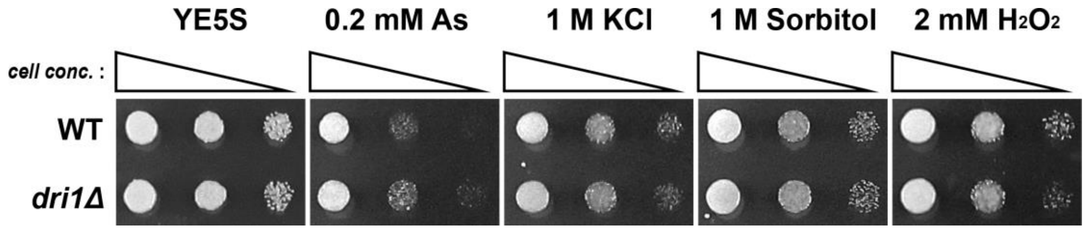

Supplementary Figure S7. Sensitivity/resistance of the *dri1* mutants is not altered under various stress conditions besides high temperature. Indicated strains were serially (10-fold) diluted, spotted onto rich YE5S plates that contains various reagents and incubated at various temperatures for 2–3 d.

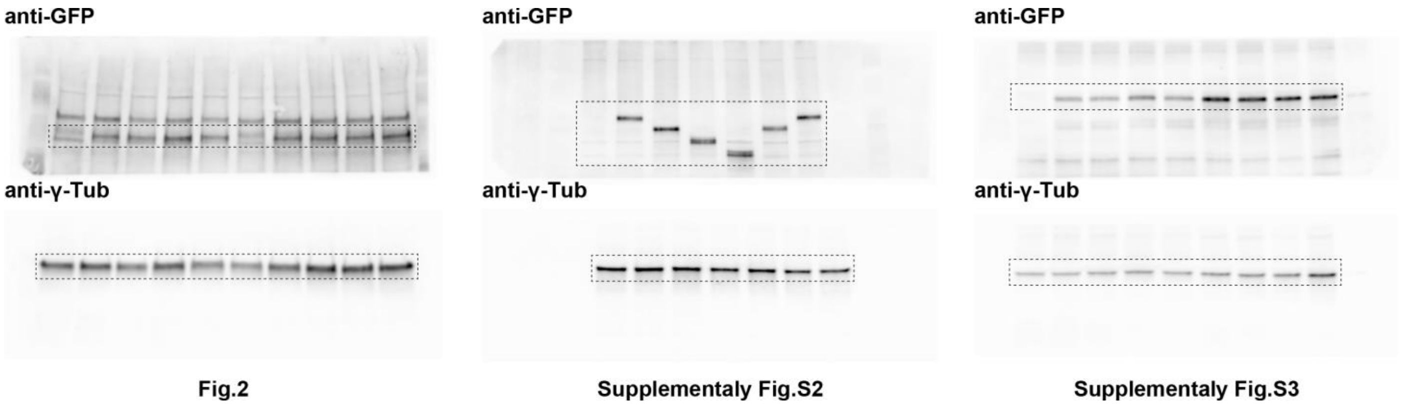

Supplementary Figure S8. Full scans of original images.

Supplementary Table S1. Fission yeast strains used in this study

Strains	Genotypes	Figures used	Derivations
513	*h*$^-$ *leu1 ura4*	1C, 2D, 4D, 6C, S1, S2, S3A, S7	Our lab stock
YY146	*h*$^+$ *cut7-22 leu1ura4 his2*	1C, 4D, S1	Our lab stock
YY50	*h*$^+$ *cut7-22 dri1-W46X leu1ura4 his2*	1C	This study
MY1479	*h*$^+$ *cut7-22 dri1::kanR leu1 ura4 his2*	1C, S1	This study
MY2087	*h*$^-$ *cut7-22 dri1Δ100C-GFP-kanR leu1 ura4*	1C	This study
MY2106	*h*$^-$ *cut7-22 dri1Δ200C-GFP-kanR leu1 ura4*	1C	This study
MY2108	*h*$^-$ *cut7-22 dri1Δ278C-GFP-kanR leu1 ura4*	1C	This study
MY2089	*h*$^-$ *cut7-22 dri1ΔRRM-GFP-kanR leu1 ura4*	1C	This study
MY2135	*h*$^-$ *cut7-22 dri1-F290A-GFP-kanR leu1 ura4*	1C	This study
MY1744	*h*$^-$ *kanR-Palp4-GFP-klp2 aur1::aur1R-Pnda3-mCherry-atb2 leu1 ura4*	2A-D, 5C	This study
MY1746	*h*$^+$ *cut7-22 kanR-Palp4-GFP-klp2 aur1::aur1R-Pnda3-mCherry-atb2 leu1 ura4 his2*	2A-D	This study
MY1747	*h*$^+$ *cut7-22 dri1::kanR kanR-Palp4-GFP-klp2 aur1::aur1R-Pnda3-mCherry-atb2 leu1 ura4 his2*	2A-D	This study
MY1745	*h*$^-$ *dri1::kanR kanR-Palp4-GFP-klp2 aur1::aur1R-Pnda3-mCherry-atb2 leu1 ura4*	2B-D	This study
MiO222	*h*$^?$ *dri1-GFP-KanR sid4-mRFP-natR aur1::aur1R-Pnda3-mCherry-atb2 leu1 ura4*	3A	This study
MY2170	*h*$^-$ *dri1-GFP-kanR hht1-mCherry-kanR leu1 ura4*	3B-C	This study
MY2171	*h*$^+$ *dri1Δ100C-GFP-kanR hht1-mCherry-kanR leu1 ura4*	3B-C	This study
MY2174	*h*$^+$ *dri1Δ200C-GFP-kanR hht1-mCherry-kanR leu1 ura4 his2*	3B-C	This study
MY2175	*h*$^+$ *dri1Δ278C-GFP-kanR hht1-mCherry-kanR leu1 ura4 his2*	3B-C	This study
MY2177	*h*$^+$ *dri1ΔRRM-GFP-kanR hht1-mCherry-kanR leu1 ura4 his2*	3B-C	This study
MY2178	*h*$^-$ *dri1-F290A-GFP-kanR hht1-mCherry-kanR leu1 ura4*	3B-C	This study
MY2467	*h*$^-$ *dri1-mCherry-hphR hsp104-GFP-ura4*$^+$ *leu1? ura4?*	4A	This study
MY2477	*h*$^-$ *dri1-mCherry-hphR ssa1-GFP-natR leu1? ura4?*	4A	This study
MY2434	*h*$^+$ *dri1-mCherry-hphR sty1′-GFP-ssa2-leu1+ leu1 ura4*	4A	This study
MY2491	*h*$^+$ *dri1-mCherry-hphR sty1′-GFP-mas5-leu1*$^+$ *leu1? ura4? his2*	4A	This study

MY2473	h^- *dri1-mCherry-hphR eIF4E-GFP-natR leu1? ura4?*	4A	This study
MY1799	h^- *dri1-mNeonGreen-hphR leu1 ura4*	4B	This study
MY2520	h^- *dri1-mNeonGreen-hphR hsp104::kanR leu1 ura4*	4B	This study
MY2526	h^- *dri1-mNeonGreen-hphR psi1::ura4*$^+$ *leu1 ura4*	4B	This study
MY2536	h^- *dri1-mNeonGreen-hphR mas5::kanR leu1 ura4*	4B	This study
MY2532	h^- *hsp104-GFP-ura4*$^+$ *dri1::kanR leu1? ura4?*	4C	This study
MY2528	h^- *sty1′-GFP-mas5-leu1*$^+$ *dri1::kanR leu1 ura4?*	4C	This study
MY2413	h^+ *hsp104::kanR ura4 leu1 his2*	4D	This study
MY2425	h^- *psi1::ura4*$^+$ *ura4? leu1*	4D	This study
MY2544	h^+ *mas5::kanR leu1? ura4 his2*	4D	This study
MY2415	h^+ *cut7-22 hsp104::kanR leu1 his2*	4D	This study
MY2427	h^+ *cut7-22 psi1::ura4*$^+$ *ura4? leu1 his2*	4D	This study
MY2551	h^+ *cut7-22 mas5::kanR leu1? ura4 his2*	4D	This study
MY2698	h^+ *cut7-22 dri1-GBP-6HIS-mCherry-hphR sty1′-GFP-ssa2-leu1*$^+$ *leu1? ura4?*	5A-B	This study
MY2669	h^- *dri1-GBP-6HIS-mCherry-hphR leu1? ura4?*	5B	This study
MY2623	h^+ *cut7-22 dri1-GBP-6HIS-mCherry-hphR leu1 ura4 his2*	5B	This study
MY2743	$h^?$ *cut7-22 sty1′-GFP-ssa2-leu1*$^+$ *aur1::aur1R-Pnda3-mCherry-atb2 leu1? ura4?*	5C	This study
MY2744	$h^?$ *cut7-22 dri1-GBP-6HIS-mCherry-hphR aur1::aur1R-Pnda3-mCherry-atb2 leu1? ura4?*	5C	This study
MY2746	$h^?$ *cut7-22 dri1-GBP-6HIS-mCherry-hphR aur1::aur1R-Pnda3-mCherry-atb2 leu1? ura4?*	5C	This study
MiO99	h^- *dri1-GFP-kanR leu1 ura4*	6A, S2, S4A	This study
MY1978	h^- *dri1-mNeonGreen-hphR pabp-mRFP-hphR leu1 ura4*	6B	This study
MiO233	h^+ *dri1-mNeonGreen-hphR dcp1-mCherry-natR leu1 ura4 his2*	6B	This study
MiO77	h^+ *dri1::kanR leu1 ura4 his2*	6C, S1, S7	This study
MY2064	h^- *dri1Δ100C-GFP-kanR leu1 ura4*	6C, S2	This study
MY2066	h^- *dri1Δ200C-GFP-kanR leu1 ura4*	6C, S2	This study
MY2068	h^- *dri1Δ278C-GFP-kanR leu1 ura4*	6C, S2	This study
MY2691	h^- *dri1ΔRRM-GFP-kanR leu1 ura4*	6C, S2	This study
MY2120	h^+ *dri1-F290A-GFP-kanR leu1 ura4 his2*	6C, S2	This study
MY1008	h^- *cut7-21 leu1 ura4*	S1	Our lab stock
MiO107	h^- *cut7-21 dri1::kanR leu1 ura4*	S1	This study
MA2-3D	h^- *cut7-23 leu1*	S1	Our lab stock
MiO108	h^- *cut7-23 dri1::kanR leu1 ura4*	S1	This study
IH136	h^- *cut7-24 leu1*	S1	Our lab stock
MiO109	h^- *cut7-24 dri1::kanR leu1 ura4*	S1	This study
NK193	h^+ *cut7-446 leu1 his2*	S1	Our lab stock
MiO110	h^+ *cut7-446 dri1::kanR leu1 ura4 his2*	S1	This study
MA145	h^- *mal3-GFP-kanR leu1 ura4*	S3A-B	Our lab stock
MY2247	h^- *mal3-GFP-kanR dri1::natR leu1 ura4*	S3A-B	This study
MY2264	h^- *cut7-22 mal3-GFP-kanR leu1 ura4*	S3A	This study
MY2266	h^- *cut7-22 dri1::natR mal3-GFP-kanR leu1 ura4*	S3A	This study
MiO190	h^- *rae1-167 dri1-GFP-kanR leu1? ura4*	S4A	This study
MY2741	h^- *rae1-167 dri1-GFP-kanR hht1-mCherry-kanR leu1? ura4*	S4B	This study
MY2475	h^- *dri1-mCherry-hphR hsp16-GFP-natR leu1? ura4?*	S5	This study
MY2471	h^- *dri1-mCherry-hphR sty1′-GFP-psi1-leu1*$^+$ *ura4?*	S5	This study
MY2469	h^+ *dri1-mCherry-hphR swo1-GFP-natR leu1? ura4? his2*	S5	This study
MY1281	h^- *cut12-GFP-ura4*$^+$ *aur1::aur1R-Pnda3-mCherry-atb2 leu1 ura4*	S6A-B, S6D	This study
MY2757	h^- *dri1::kanR cut12-GFP-ura4*$^+$ *aur1::aur1R-Pnda3-mCherry-atb2 leu1 ura4*	S6A-B, S6D	This study
MY2759	h^- *dri1::kanR hht1-mCherry-kanR rlc1-GFP-ura4*$^+$ *leu1 ura4*	S6C	This study

*Strains were developed for this study unless otherwise specified. *his2=his2-245*; *leu1=leu1-32*; *ura4=ura4-D18*.

Copyright: © 2021 by the author. Licensee MDPI, Basel, Switzerland. This article is an open access article distributed under the terms and conditions of the Creative Commons Attribution (CC BY) license (http://creativecommons.org/licenses/by/4.0/).
